# Identification and Validation of a Novel 2-LncRNAs Signature Associated with m6A Regulation in Colorectal Cancer

**DOI:** 10.7150/jca.64817

**Published:** 2022-01-01

**Authors:** Kangchun Wang, Bei Zhao, Yu Liang, Bin Ma

**Affiliations:** 1 Department of Organ Transplantation and Hepatobiliary, The First Affiliated Hospital of China Medical University, No.155 Nanjing North Street, Heping District, Shenyang 110001, Liaoning Province, China.; 2 Department of Ultrasound, Xiang'an Hospital of Xiamen University, No.2000 Xiang'an East Road, Xiang'an District, Xiamen 361101, Fujian Province, China.; 3 Department of Colorectal Surgery, Cancer Hospital of China Medical University, Liaoning Cancer Hospital and Institute. No.44 Xiaoheyan Road, Dadong District, Shenyang 110042, Liaoning Province, China.

**Keywords:** colorectal cancer, N6-methyladenosine, biomarker, lncRNA signature, prognostic model.

## Abstract

Colorectal cancer (CRC) is one of the most common tumors in the digestive system, and it is urgent to identify a new biomarker for the diagnosis and treatment of CRC. N6-methyladenosine (m6A) is an abundant mRNA modification and is almost involved in every aspect of physiological processes. In this study, we constructed a novel m6A-related 2-lncRNAs signature that can predict the prognosis of CRC. We obtained m6A-related lncRNAs and identified prognostic lncRNAs through univariate Cox regression analysis and least absolute shrinkage and selection operator (LASSO) analysis, then constructed a prognostic model based on the risk score, and we also verified the stability of the model. In addition, differential expression analysis between the high- and low-risk subgroups was performed. A total of 1,894 m6A-related lncRNAs were screened from various sources. Using univariate Cox regression analysis and survival analysis, two lncRNAs (AL135999.1 and AL049840.4) were identified (*P* < 0.05), and the coefficients of lncRNAs were calculated by LASSO. The high-risk group had worse clinical outcomes and overall survival (OS) than the low-risk group, and the risk score can serve as an independent prognostic factor in CRC. In addition, different stages of CRC also showed a different level of risk score. Finally, we found that two lncRNAs were differentially expressed (*P* < 0.01) in CRC patients, and AL135999.1 may be relevant to m6A modification mediated by methyltransferase-like 3 (METTL3) in CRC. In summary, we constructed a reliable 2-lncRNAs signature based on the risk score, and we identified two m6A-related prognostic lncRNAs, AL135999.1 and AL049840.4. The novel 2-lncRNAs signature plays an essential role in predicting the prognosis of CRC.

## Introduction

Colorectal cancer (CRC) is one of the most common malignancies of the digestive system 1. Although CRC can be treated with standard strategies such as surgery, radiation therapy, and chemotherapy, nearly half of patients present with metastasis at the time of diagnosis or exhibit resistance during treatment 2. Therefore, new molecular targets are urgently needed to improve the diagnostic efficacy and individual treatment response.

N6-methyladenosine (m6A) RNA modification, the most abundant mRNA modification, was first discovered in 1974 3. It is involved in almost every aspect of mRNA metabolism and various physiological processes 4. A growing number of researches indicate that m6A modification is closely related to the carcinogenesis and metastasis of CRC. Some researchers suggest that the methyltransferase-like 3 (METTL3)-catalyzed m6A modification plays a critical role in the occurrence of CRC, wherein it facilitates tumor growth by suppressing YPEL5 expression in an m6A-YTHDF2-dependent manner 5. The m6A modification mediated by methyltransferase-like 14 (METTL14) was significantly downregulated in CRC, resulting in CRC patients' poor overall survival (OS) rate. This phenomenon occurs because SRY-related high-mobility-group box 4 (SOX4) is a target of METTL14-mediated m6A modification, which affects the epithelial-mesenchymal transition (EMT) process in the tumor and leads to the metastasis of CRC 6. In recent years, long noncoding RNAs (lncRNAs) have received continuous attention. LncRNAs participate in the signal transduction pathway of CRC, interaction of various DNA and RNA molecules, and the process of tumorigenesis 7. It has been reported that the lncRNA X inactivate-specific transcript (XIST) is the downstream target of METTL14. Knockdown of METTL14 can eliminate the m6A and increase the expression of XIST, which can dramatically increase the proliferation and invasion of CRC 8. In addition, m6A-induced lncRNA RP11 expression can trigger EMT and invasion of CRC cells via posttranslational upregulation of Zinc Finger E-Box Binding Homeobox 1 (ZEB1), which may be one of the crucial molecular mechanisms in CRC metastasis 9. However, studies on m6A-related lncRNAs in CRC are still few and unclear. The underlying molecular mechanisms need further investigation, and it provides a more promising strategy to prevent, diagnose, and treat CRC.

In this study, after univariate Cox regression analysis and least absolute shrinkage and selection operator (LASSO) analysis, we screened two lncRNAs (AL049840.4 and AL135999.1) in The Cancer Genome Atlas (TCGA) database. Subsequently, we developed a novel 2-lncRNA prognostic biomarker associated with m6A in CRC, and the predictive performance of the biomarker was tested with the receiver operating characteristic (ROC) curve. We evaluated the relationship between the risk score and clinical characteristics, and we suggested that the risk score can serve as an independent prognostic factor in CRC. Based on the risk score and differential expression analysis, we performed enrichment analysis on the differentially expressed genes (DEGs), and we explored the expression level of m6A-related genes in different subgroups. Finally, we validated that AL135999.1 and AL049840.4 are differentially expressed between CRC and normal tissues, and AL135999.1 may be relevant to m6A modification mediated by METTL3 in CRC.

## Materials and methods

### Data source

We downloaded the RNA-seq data [Fragments Per Kilobase of transcript per Million mapped reads (FPKM) normalized data] and clinical information from the TCGA database. In order to reduce the statistical bias in our study, we excluded all patients with missing OS values. Finally, we obtained 618 CRC samples in the downloaded datasets. The m6A2Target database is an open-access website for searching m6A target genes 10. We obtained m6A-related genes from the m6A2Target database and related literature and then searched for m6A-related lncRNAs based on these genes. m6A-related lncRNAs have three origins in the present study: We implemented Pearson correlation analysis between m6A-related genes and lncRNAs with the thresholds *P* < 0.01 and |R| > 0.6. In addition, we identified m6A-related lncRNAs based on the m6A2Target database and starBase database 11.

### Univariate Cox regression and LASSO Cox regression analysis

To identify m6A-related lncRNAs with prognostic value, we performed the univariate Cox regression analysis with the threshold of *P* < 0.05. All lncRNAs meeting the threshold criterion were used for further survival analysis. We implemented the LASSO analysis by the R package "glmnet" 12 and constructed a risk model that can predict the prognosis of CRC patients. The risk score was calculated based on the coefficients of lncRNAs, and the formula is:



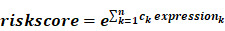



where 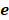 is the natural constant, 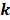 is the
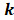
th lncRNA with a non-zero coefficient, 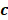 is the coefficient, and 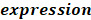 is the FPKM value of the lncRNA.

### The prognostic model and ROC curve

According to the risk score calculated in the previous step, we randomly divided the TCGA samples into training and validation cohorts. Based on the median risk score, all the samples were divided into high- and low-risk groups, and we visualized the scatter plot and Kaplan-Meier (K-M) curves to describe the distribution of risk score and clinical outcomes by the R package "survival". In addition, the ROC curves were used to evaluate the ability to predict OS in the TCGA cohort (1-year, 3-year, and 5-year), the area under the ROC curve serves as the evaluation standard.

### Validation of independent prognostic factor and clinical characteristics

We sought to determine whether the clinical characteristics were associated with the risk score, so we divided the samples into different groups according to clinical information (sex, age, race, and stage) and assessed whether the risk scores were different within each group. Then, we conducted univariate and multivariate Cox regression analyses to verify whether the risk score can serve as an independent prognostic factor.

### Differential expression analysis and enrichment analysis

We carried out differential expression analysis and enrichment analysis between high- and low-risk groups of TCGA samples, which were divided by the median risk score. The DEGs were identified with the threshold of *FDR* < 0.05 and |log2FC| > 1 using the R package "limma" 13. We also attempted to identify the main biological functions and processes of DEGs, enrichment analysis and networks of enriched terms performed in the Metascape 14, including Gene Ontology (GO) Biological Processes, Kyoto Encyclopedia of Genes and Genomes Pathway (KEGG), Canonical Pathways, and Reactome Gene Sets. Then, we studied the expression of m6A-related genes in the high- and low-risk groups and identified significant genes according to the *P*-value.

### Clinical tissues samples

A total of 50 tissues from CRC patients with paired nontumor adjacent tissues (NATs) obtained via surgical resection were provided by Liaoning Cancer Hospital & Institute of China Medical University (Shenyang, Liaoning, China). The investigation was approved by the Institute Research Ethics Committee of Liaoning Cancer Hospital of China Medical University and conducted in accordance with the Declaration of Helsinki.

Inclusion criteria: (1) The first primary tumor; (2) The patient was diagnosed with pathological confirmation; (3) The patient has not received chemotherapy, radiotherapy, or target therapy before the surgery; (4) Age of 18 or older; (5) Without distant metastasis. Exclusion criteria: (1) The patient with missing pathological data or unclear diagnosis; (2) The patient has received other treatments before the surgery.

### Cell culture

A total of 5 human colorectal cancer cell lines were used in this study, including HCT116, HT29, RKO, SW620, SW480, and normal colonic epithelial cell line (FHC). All cell lines were purchased from the American Type Culture Collection. Cells were cultured in the RPMI medium (Hyclone, Logan, United States) supplemented with 10% fetal bovine serum, and cells were maintained in a 37°C incubator with 5% CO_2_.

### Quantitative real‐time PCR (qRT‐PCR) analysis

Based on the manufacturer's protocols, we extracted total RNA from tissues using TRIzol reagent (Invitrogen, Carlsbad, United States), and we used PrimeScriptTM RT reagent Kit (Takara, Otsu, Japan) to reverse transcribe total RNA. All reactions were conducted in triplicate for each sample, and the expression levels were calculated by using the 2^-△△Ct^ method. The process was conducted on a Light Cycler 480 II Real‐Time PCR system (Roche Diagnostics, Rotkreuz, Switzerland), and the qRT‐PCR cycle was repeated 45 times with the conditions of 95°C, 60°C and 72°C for 5, 20 and 30 seconds respectively. The primer sequences are described in **Table S1.**

### RNA immunoprecipitation (RIP)

The Magna RIP RNA Binding Protein Immunoprecipitation Kit (Millipore, Bedford, United States) was used for RIP experiments by the manufacturer's protocol. The complete RIP lysis buffer was used to lyse the cells in this experiment. Then, we incubated the cell extracts with protein A/G agarose beads bound to specific antibodies or control IgG under appropriate conditions, and then the beads were washed and incubated with proteinase K to remove protein. We then eluted the immunoprecipitated RNA and analyzed the RNA by qRT-PCR.

### Statistical analysis

Statistical analysis was performed using SPSS 24.0 (IBM Corporation, Armonk, USA) and R programming language (version 4.0.2). Pearson correlation was used to analyze the correlation between the m6A genes and lncRNAs, and we constructed the Kaplan-Meier curves and conducted a log-rank test to compare the OS between different subgroups. The ROC curves and the area under the curve (AUC) value are performed to evaluate the OS prediction ability of the model in 1/3/5 years. Finally, univariate and multivariate Cox regression analyses are used to test whether risk score can serve as an independent predictor. The t-test determines the difference between groups. *P* <0.05 is considered to indicate a statistically significant difference.

## Results

### Identification of m6A-Related lncRNAs

**Figure 1:**
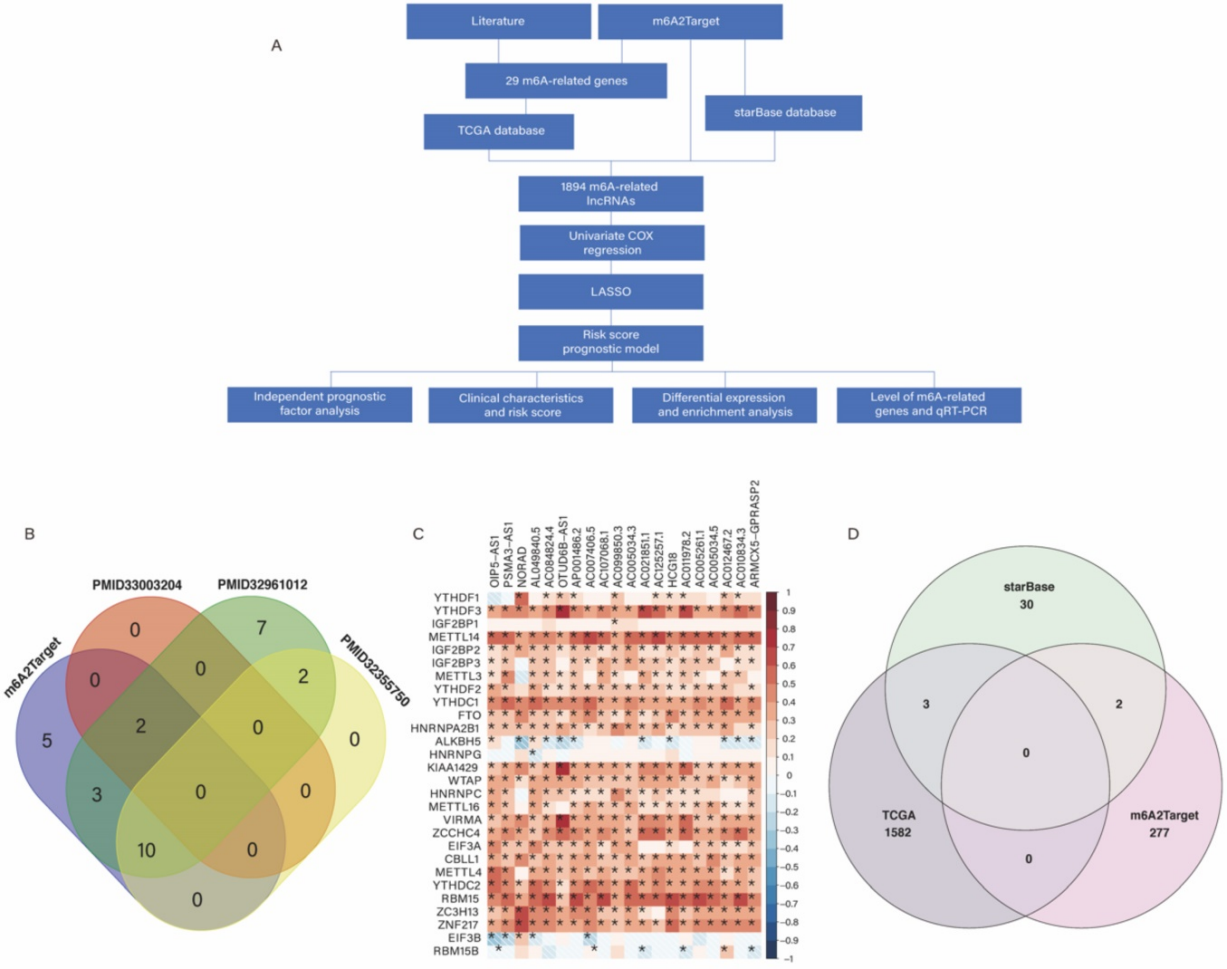
** (A)** Workflow of this study. **(B)** Venn plots showing the source of m6A-related genes.** (C)** Heatmap of the correlations between m6A-related genes and the top 20 m6A-related lncRNAs. *P < 0.05. **(D)** Venn plots showing the source of m6A-related lncRNAs.

Based on our workflow **(Figure 1A)**, a total of 29 m6A-related genes were identified, of which 20 were obtained from the m6A2Target database, 2 from PMID33003204 15, 24 from PMID32961012 16, and 12 from PMID32355750 17** (Figure 1B)**. There were 622 CRC samples in the downloaded TCGA datasets, of which 618 samples contained clinical information, as shown in **Figure S1.** According to the results of Pearson analysis, 1585 lncRNAs were obtained from the TCGA datasets, the heatmap of the top 20 m6A-related lncRNAs is displayed (*P* < 0.05) **(Figure 1C)**. In addition, we identified 279 and 35 m6A-related lncRNAs in the m6A2Target database and starBase database respectively, and constructed the m6A-related lncRNAs networks shown in **Figure S2.** In short, we identified a total of 1,894 lncRNAs from three origins **(Figure 1D)**.

### Univariate Cox regression and LASSO analysis

**Figure 2:**
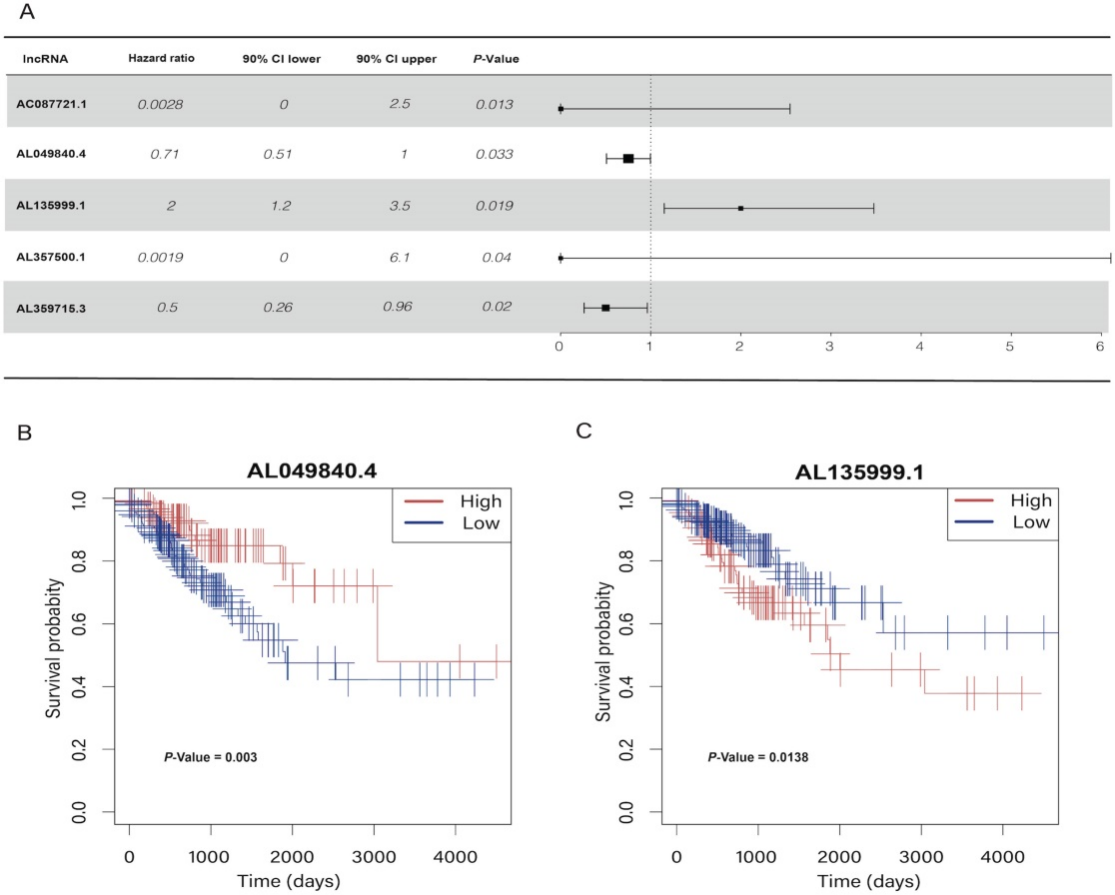
Univariate Cox regression analysis and Kaplan-Meier curves.** (A)** Univariate Cox regression analysis indicated that five genes play a critical role in the prognosis of colorectal cancer. **(B, C)** Kaplan-Meier curves showed that patients in high- and low-expression groups of AL049840.4 and AL135999.1 had different overall survival.

**Figure 3:**
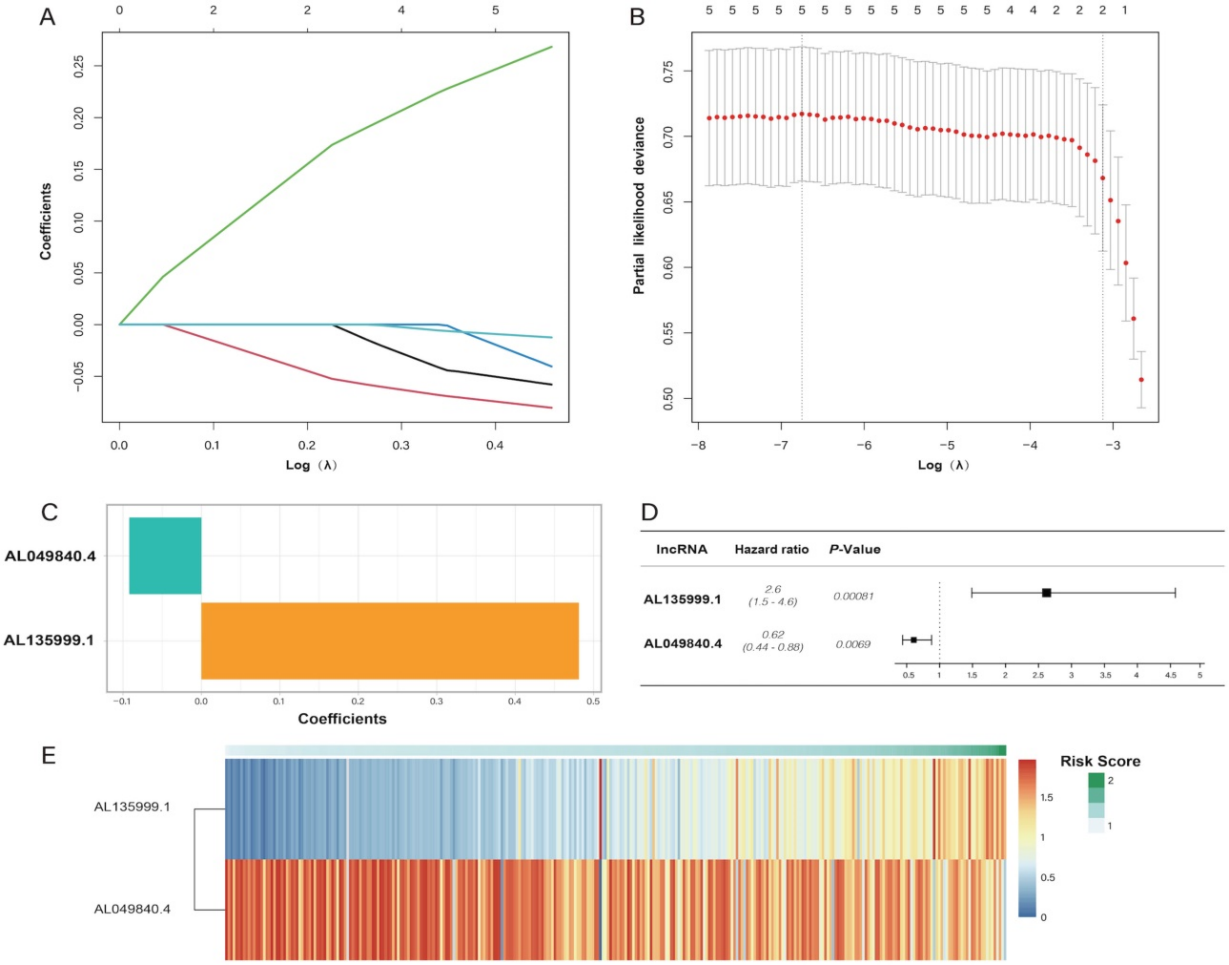
Least absolute shrinkage and selection operator (LASSO) regression analysis. **(A, B)** showing the calculation of minimum criteria. **(C)** showing the coefficients. The forest plot of AL135999.1 and AL049840.4 is displayed in **(D)**, and the heatmap of patients' risk scores is displayed in** (E)**.

In the univariate Cox regression results, we identified five m6A-related lncRNAs (AC087721.1, AL049840.4, AL135999.1, AL357500.1, and AL359715.3) that were significantly correlated with the prognosis of CRC patients (*P* < 0.05) **(Figure 2A)**, and then we visualized K-M curves of these lncRNAs. The OS of AL049840.4 and AL135999.1 were significantly different between high and low expression groups. Upregulation of AL049840.4 was a protective factor, and the high expression group had better clinical outcomes (*P* = 0.003), in contrast to the group with upregulation of AL135999.1, which served as a risk factor (*P* = 0.0138)** (Figure 2B, C)**. Subsequently, LASSO analysis was performed to generate coefficients of m6A-related lncRNAs, and the results indicated that the coefficients of the lncRNAs AL049840.4 (coefficient = -0.09195067) and AL135999.1 (coefficient = 0.48118056) contributed to the risk model **(Figure 3)**.

### Construction of the prognostic model

**Figure 4:**
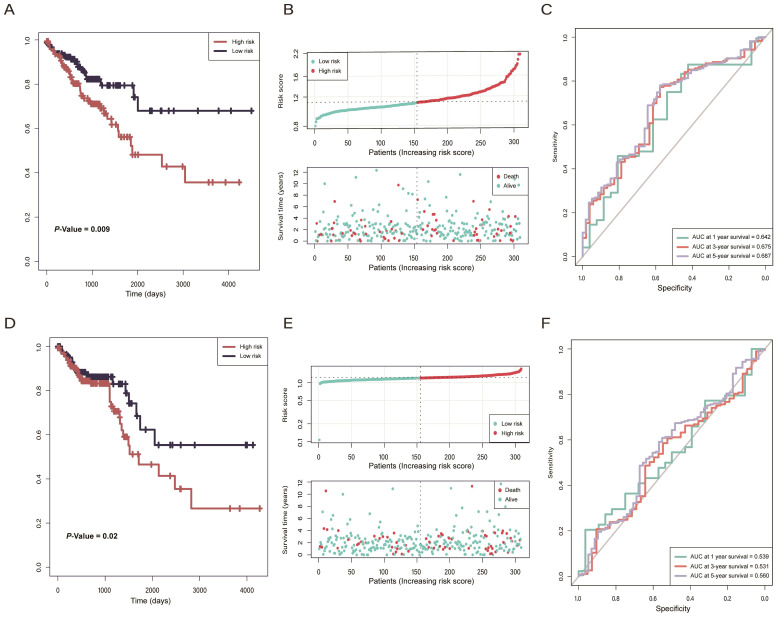
** (A)** Kaplan-Meier curves of The Cancer Genome Atlas (TCGA) training cohort indicated that patients in the high-risk group had a worse overall survival rate than the low-risk group. **(B)** The distribution of the risk score and status of colorectal cancer (CRC) patients in TCGA training cohort. **(C)** ROC curves of prognostic ability within 1/3/5-year in TCGA training cohort. **(D)** Kaplan-Meier curves of the TCGA validation cohort indicated that patients in the high-risk group had a worse overall survival rate than the low-risk group. **(E)** The distribution of the risk score and status of CRC patients in the TCGA validation cohort. **(F)** ROC curves of prognostic ability within 1/3/5-year in TCGA validation cohort.

According to the risk model of 2-lncRNAs based on risk score, we divided the samples into high- and low-risk groups and then analyzed the distribution and clinical outcomes of patients. The results showed that the OS was significantly different between high- and low-risk groups (*P* = 0.009). Comparison of the two groups indicated that patients in the high-risk group had lower survival rates and shorter OS times, which led to poor clinical outcomes **(Figure 4A, B)**. In ROC curve analysis, the AUC reached 0.6 in 1-year (AUC = 0.642), 3-year (AUC = 0.675), and 5-year (AUC = 0.687). **(Figure 4C)**, which indicates that the 2-lncRNAs risk model is reliable and has the potential for predicting the prognosis of CRC in the TGCA datasets. In the validation cohort, there were significantly different survival outcomes between the high- and low-risk groups (*P* = 0.02), and the high-risk group tended to have a shorter survival time **(Figure 4D, E)**. In the ROC curve, the AUC reached 0.56 in 5-year **(Figure 4F)**.

### The risk score is an independent prognostic factor

**Figure 5:**
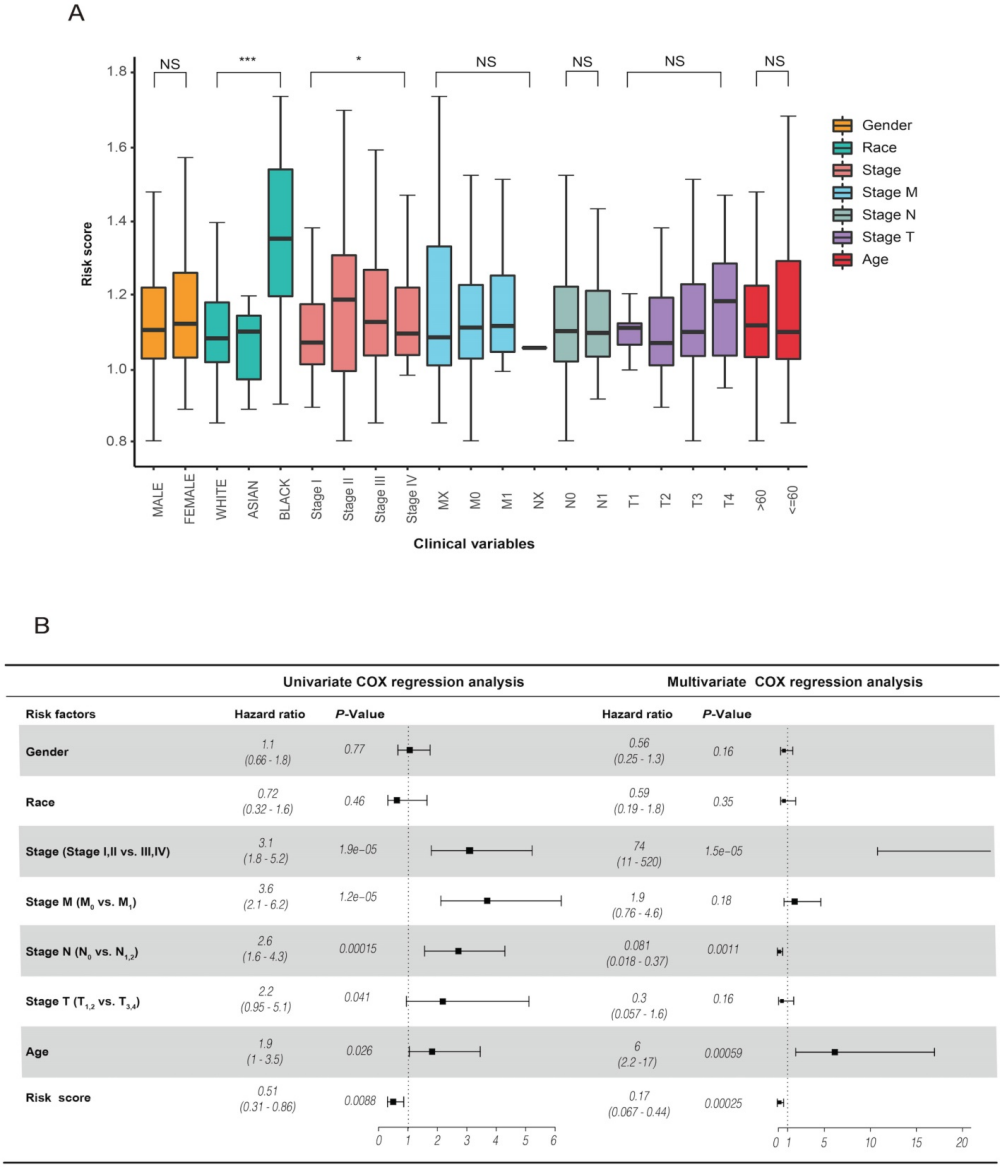
** (A)** revealed the relationship between risk score and clinical characteristics. **(B)** Univariate and multivariate analyses indicated that the risk score was an independent prognostic predictor in the TCGA datasets.

The analysis of the risk score and clinical characteristics showed that there were obvious differences in "race" and "stage" between the high- and low-risk groups in CRC **(Figure 5A)**. In addition, we found that the risk score was highly associated with OS in univariate Cox regression analysis [HR = 0.51, 90% CI: 0.31-0.86, *P* = 0.0088]. Using multivariate Cox regression analysis, we identified that the risk score was an independent prognostic factor for CRC patients [HR = 0.17, 90% CI: 0.067-0.44, *P* = 0.00025] **(Figure 5B)**. Our results suggested that the risk score is a worthy independent indicator for evaluating the prognosis of CRC.

### Identification of DEGs and enrichment analysis

**Figure 6:**
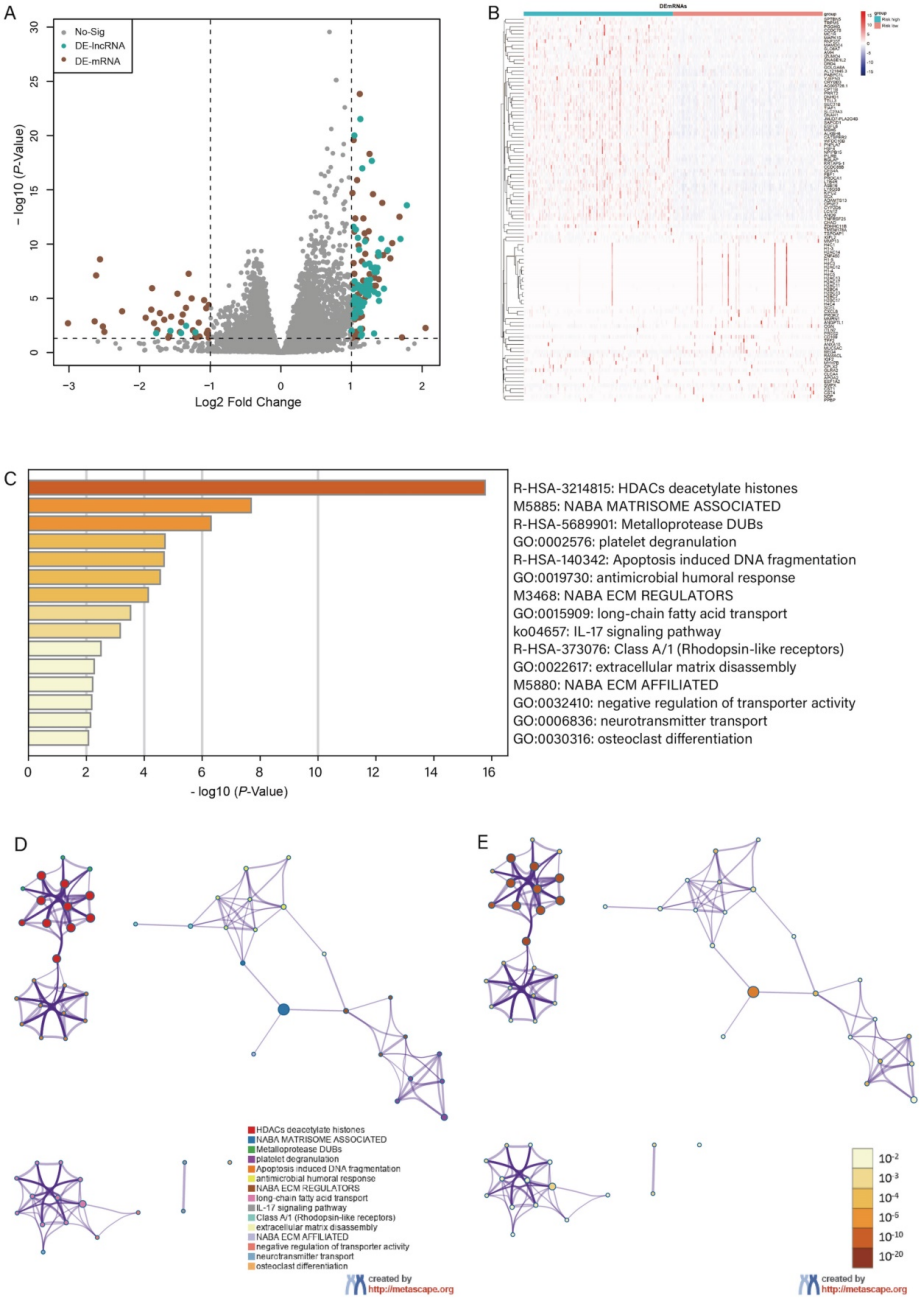
Differential expression and enrichment analysis. Volcano plot of differential expression analysis displayed in **(A)** and the heatmap of differential expressed genes displayed in **(B)**. **(C)** Histogram of enrichment terms colored according to the* P*-value. We constructed an enriched term network based on the cluster-ID **(D)** and *P*-value** (E)**.

**Figure 7:**
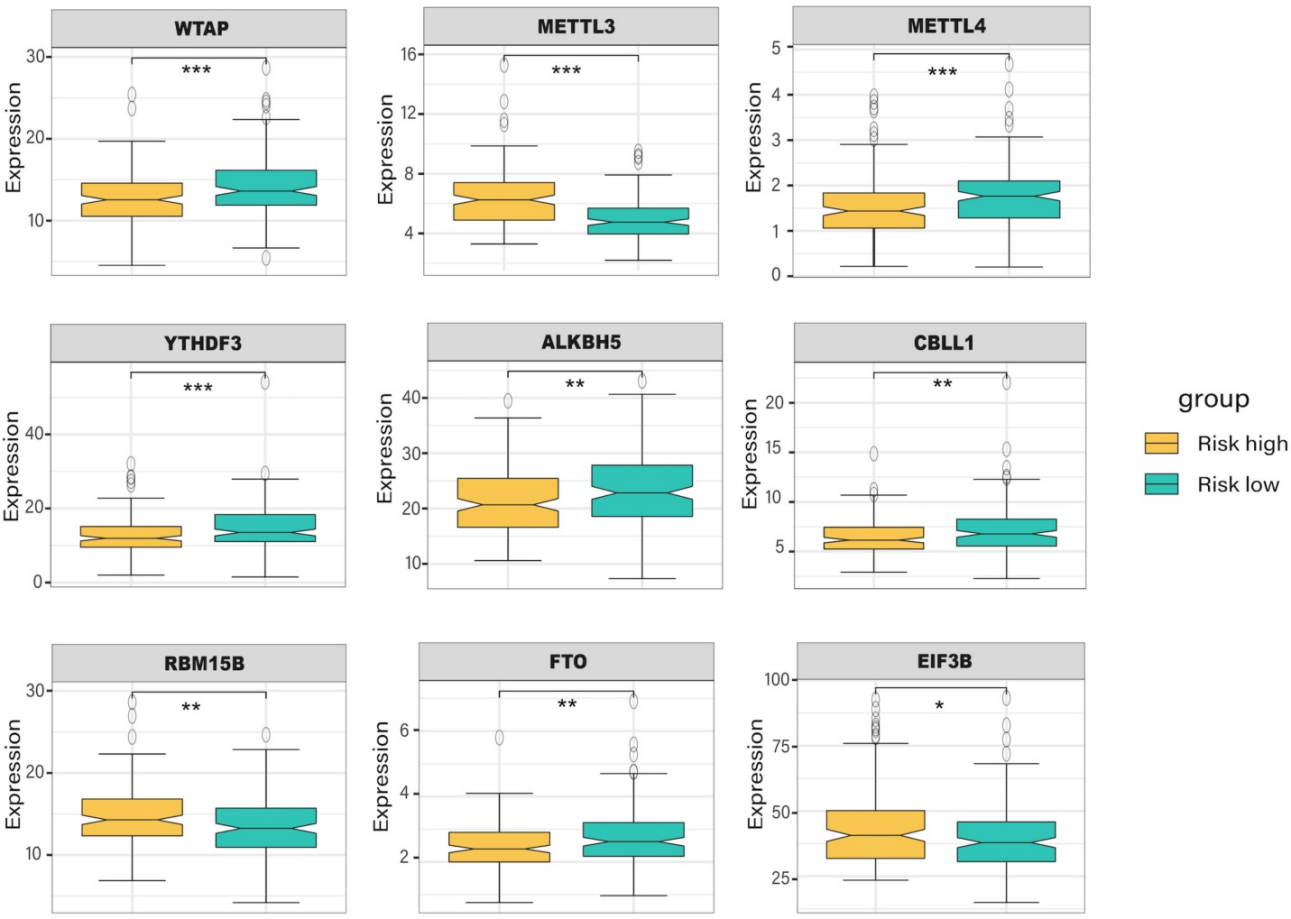
The expression level of m6A-related genes in the high- and low-risk group of colorectal cancer patients.

With the criteria of *FDR* < 0.05 and |log2FC| > 1, we identified 104 differentially expressed genes between high- and low-risk groups, and the volcano plot and the clustering heatmap of DEGs were visualized **(Figure 6A, B)**. In the enrichment analysis, we found that the DEGs were mainly enriched in apoptosis-induced DNA fragmentation, metalloproteinase DUB, HDACs deacetylate histones (Reactome gene sets); NABA matrisome-associated, NABA ECM modulator (canonical pathways); and antimicrobial humoral response, long-chain fatty acid transport, and platelet degranulation (GO biological processes) **(Figure 6C)**. In the network of enriched terms, nodes with the same cluster-ID and *P*-value were closer to each other** (Figure 6D, E)**, and comprehensive enrichment analysis enabled us to gain a deep understanding of the biological processes in which these DEGs are involved. Finally, we found that some m6A-related genes were differentially expressed between the high- and low-risk groups **(Figure 7)**, especially WTAP, METTL3, METTL4, and YTHDF3 (*P* < 0.001). These DEGs may serve as potential molecular targets in the precise diagnosis and treatment of CRC in the future.

### Validation in CRC tissues and cell lines

**Figure 8:**
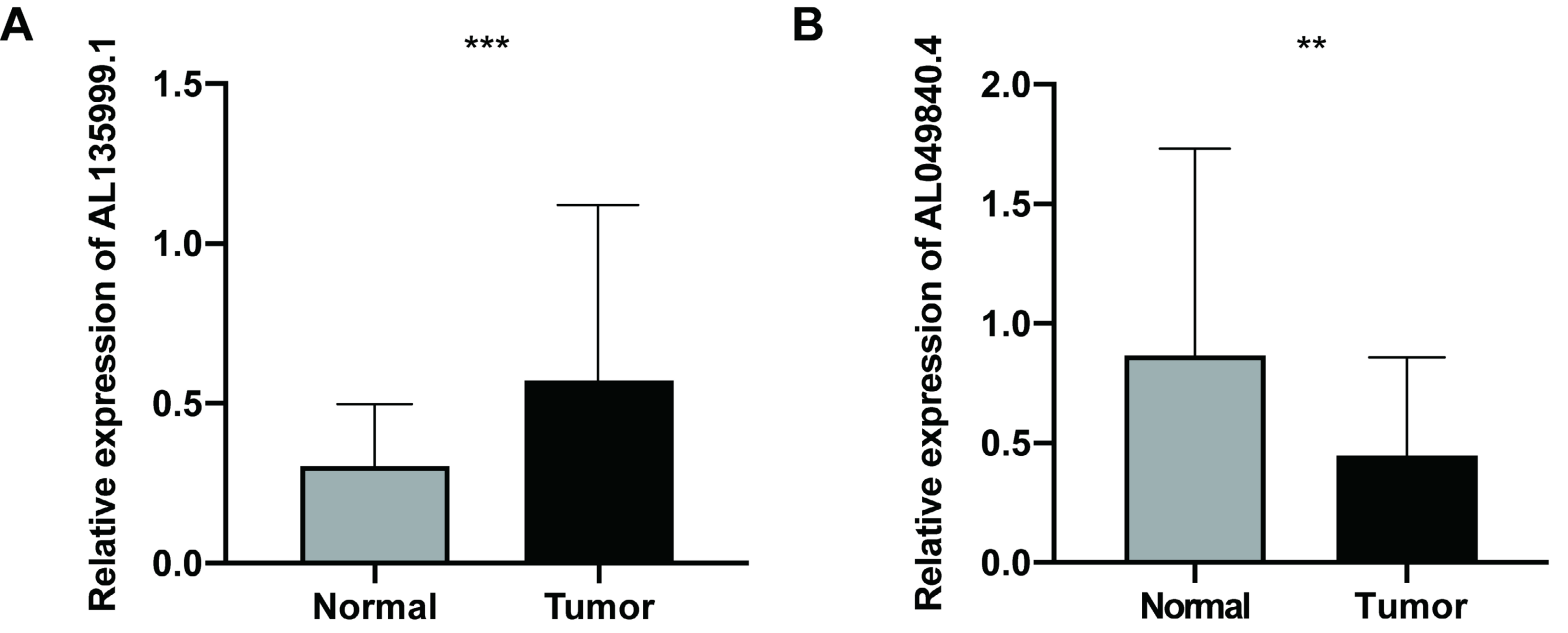
The expression levels of lncRNAs (AL135999.1 and AL049840.4) in colorectal cancer (CRC). **(A)** The relative expression levels of AL135999.1 between CRC tissues and nontumorous adjacent tissues (NATs).** (B)** The relative expression levels of AL049840.4 between CRC tissues and NATs.

Compared with the NATs by qRT‐PCR analysis, the lncRNA AL135999.1 was significantly upregulated in CRC tissues (*P* < 0.01), and AL049840.4 was significantly downregulated (*P* < 0.01) **(Figure 8)**. This finding was consistent with our previous bioinformatics analysis results. METTL3 is an m6A methyltransferase that is essential for the development of CRC 5, 18, 19, and the level of AL135999.1 was upregulated in CRC cell lines **(Figure S3A)**. On this basis, we tried to explain the relationship between m6A modification and AL135999.1 in CRC. We found that m6A was highly enriched within AL135999.1 in SW480 and HCT116 cells** (Figure S3B)**. In order to clarify the correlation between the upregulation of AL135999.1 and METTL3 in CRC, we knocked down the expression of METTL3 in SW480 and HCT116, which resulted in a lower m6A level of AL135999.1 than the control group **(Figure S3C-E)**. Finally, we treated CRC cells with actinomycin D, and we found that the knockdown of METTL3 can significantly reduce the half-life of AL135999.1 in CRC cell lines. **(Figure S3F-G).** These results indicate that AL135999.1 may be relevant to CRC through m6A modification mediated by METTL3, but more experimental explorations and direct evidence are needed in the future.

## Discussion

The m6A modification was identified as a critical posttranscriptional regulatory factor in various RNAs, including mRNAs, tRNAs, and lncRNAs 20, and it is considered involved in tumor proliferation and oncogenesis 21. Scientists have validated that lncRNAs are complex regulators in tumor pathogenesis and invasion, and it is widely recognized the m6A modification of lncRNAs can affect the occurrence and development of tumors. In considering the mechanism, the m6A modification can affect the enrichment of lncRNAs and change the stability of RNA 22. Meanwhile, m6A modification can change the structure of RNA and affect the binding of lncRNA to protein 9, 23, which may have led to changes in tumor behavior. Besides, many problems such as metastasis or drug resistance in CRC will lead to a worse prognosis 24, 25. Therefore, novel prognostic biomarkers are urgently needed to improve the diagnostic efficacy and individual treatment response in CRC, which inspired us to establish an independent and reliable m6A-related lncRNAs risk model.

In the current study, we identified 1,894 m6A-related lncRNAs in public databases. After univariate Cox regression and LASSO analysis, we found that the lncRNAs AL135999.1 and AL049840.4 can be used as key lncRNAs to predict the OS of CRC patients and we constructed the m6A-related 2-lncRNAs signature based on the risk score. The clinical outcomes of patients with high-risk scores were much worse than those in the low-risk group, and the AUC of ROC reached 0.6 for 1/3/5-year. In addition, we found that the risk score is closely related to the clinical characteristics of CRC patients (race and stage), and the risk score can serve as an independent prognostic factor in CRC. It is worth noting that in the qRT-PCR analysis, these m6A-related lncRNAs, AL135999.1 and AL049840.4, were differentially expressed between CRC tissues and NATs, and our experimental results indicate that AL135999.1 may be related to METTL3-mediated m6A modification in CRC.

As a "writer" in forming the catalytic core of the m6A methyltransferase, METTL3 is an essential catalytic subunit 26-28. METTL3 exerts a significant role in both cancer occurrence and development; it promotes the expression of a variety of proteins related to cancer and affects tumor proliferation and invasiveness through EMT 29, 30. Wang et al. suggested that METTL3 can promote tumor angiogenesis and glycolysis and serves as a major regulator of the abundant m6A modification in gastric cancer (GC) 31. In addition, some researchers studied the relationship between METTL3 expression and clinical outcomes in multiple human CRC cohorts, and they revealed that the m6A-GLUT1-mTORC1 axis is an important pathway for promoting the development of CRC 19. METTL3 is also involved in the drug resistance of CRC. M2-polarized tumor-associated macrophages can elevate the level of m6A modification mediated by METTL3 and induce oxaliplatin (OX) resistance. Modulation of METTL3-mediated m6A modification may be a potential treatment strategy and molecular target for patients with OX resistance 32.

Many tumor-related lncRNAs have been reported previously, and lncRNA has been considered to play an irreplaceable role in digestive system tumors 33. In our present study, we reported the role of AL135999.1 and AL049840.4 in CRC for the first time. A similar observation was found in a study of m6A-related lncRNA in clear cell renal cell carcinoma. Researchers have found that AL135999.1 was significantly up-regulated in clear cell renal cell carcinoma tissues compared to the NATs 34. Similarly, Yuan et al. constructed a lncRNAs prognostic model in liver cancer, and twenty-seven immune-related lncRNAs were screened by univariate Cox regression, the results show that AL049840.4 has prognostic value in liver cancer 35. In addition, Zuo et al. found that the m6A level of lncRNAs in CRC tissue was significantly higher than that in NATs and the GO and KEGG results showed that the lncRNAs modified by m6A can affect the occurrence and development of CRC by biological processes, cell composition, and molecular function 36. These results suggest that lncRNA plays a vital role in predicting tumor prognosis and clarifies the relationship between lncRNA and m6A, which is beneficial to discovering new targets and treating tumors.

Finally, we summarized and compared the lncRNA signatures of CRC in several years **(Table 1)**. In fact, the existing prognostic models still have some defects, such as low efficacy and limited biological value. These problems may be caused by the method of constructing the model, the type of lncRNAs, and the source of datasets. Many lncRNA risk models are mainly based on immune-related genes or autophagy-related genes, very few studies explored the relationship between lncRNAs and m6A modification in CRC 37*.* We constructed a reliable prognostic model based on two m6A-related lncRNAs, and it can predict the clinical outcomes of CRC patients. Among the m6A-related lncRNAs, we found two novel lncRNAs (AL135999.1 and AL049840.4). AL135999.1 is a risk factor with relatively high expression in CRC samples, and AL049840.4 is a protective factor in CRC. In addition, we also explored the association between METTL3 and AL135999.1, and METTL3 may affect the stability of AL135999.1 through m6A modification. It is undeniable that there are several limitations in this research. Our study is based on retrospective data from public databases, larger-scale prospective data ought to incorporate in the future. Moreover, the m6A modification of lncRNAs is a rather complicated process, and future research should focus on confirming the interaction between lncRNAs and m6A modification *in vitro* and *in vivo*.

## Conclusions

In summary, we identified two lncRNAs, AL135999.1 and AL049840.4, and constructed an m6A-related 2-lncRNAs signature that can predict CRC. In addition, this study provides new biomarkers for CRC patients, which may benefit further elucidating the mechanism of the occurrence and development of CRC.

## Supplementary Material

Supplementary figures and table.

## Figures and Tables

**Table 1:** The comparison of studies about lncRNA signature for CRC.

Methods	LncRNA signature	AUC Value in training cohorts			AUC Value in validation cohorts			Reference
		1-year	3-year	5-year	1-year	3-year	5-year	
Univariate and multivariate Cox regression	3-lncRNAs		0.630	0.620				Xing *et al.*
Cox regression	6-lncRNAs	0.828	0.789	0.730	0.777	0.576	0.611	Liu *et al.*
Cox regression	9-lncRNAs	0.754	0.778	0.854	0.891	0.720	0.814	Zong *et al.*
LASSO, univariate and multivariate Cox regression	6-lncRNAs	0.797	0.771		0.656	0.642		Cheng *et al.*
Multivariate Cox regression	10-lncRNAs		0.725	0.803				Sun *et al.*
LASSO regression	6-lncRNAs		0.6923	0.737		0.680	0.704	Huang *et al.*
Multivariate Cox regression	3-lncRNAs			0.716			0.649	Liu *et al.*
Univariate Cox and LASSO	3-lncRNAs		0.712	0.674		0.701	0.694	Liu *et al.*
Univariate and multivariate Cox regression	9-lncRNAs	0.768	0.778	0.870	0.761	0.801	0.883	Zhang *et al.*

## Abbreviations

CRC: colorectal cancer
m6A: N6-methyladenosine
METTL3: methyltransferase-like 3
METTL14: methyltransferase-like 14
OS: overall survival
lncRNAs: long noncoding RNAs
LASSO: least absolute shrinkage and selection operator
TCGA: The Cancer Genome Atlas
DEGs: differentially expressed genes
K-M curve: Kaplan-Meier curve
GO: Gene Ontology
KEGG: Kyoto Encyclopedia of Genes and Genomes Pathway
NATs: nontumor adjacent tissues
AUC: area under the curve
OX: oxaliplatin
XIST: X inactivate-specific transcript
ZEB1: Zinc Finger E-Box Binding Homeobox 1
SOX4: SRY-related high-mobility-group box 4
FPKM: Fragments Per Kilobase of transcript per Million mapped reads
qRT‐PCR: quantitative real‐time PCR
RIP: RNA immunoprecipitation
ROC curve: receiver operating characteristic curve
EMT: epithelial-mesenchymal transition
GC: gastric cancer
