# Supplementary material for: Identification and Validation of a Novel 2-LncRNAs Signature Associated with m6A Regulation in Colorectal Cancer
Source: J Cancer. 2022 Jan 1;13(1):21–33. doi: 10.7150/jca.64817 (PMC8692696; doi:10.7150/jca.64817)
Supplement: Supplementary file 1 — Supplementary figures and table. [file jcav13p0021s1.pdf]

Stage T				Stage N			Stage M		
T1	T2	T3	T4	N0	N1	NX	M0	M1	MX
20	105	421	33	350	109	2	458	73	64
Race				Gender		Survival		Age	
NATIVE	ASIAN	BLACK	WHITE	FEMALE	MALE	> 5 years	≤ 5 years	≤ 60	> 60
1	12	65	292	289	329	51	567	194	424
Stage									
Stage I	Stage II	Stage III	Stage IIIA	Stage IIIB	Stage IIIC	Stage IV	Stage IVA	Stage IVB	
104	37	25	15	84	55	62	24	2	

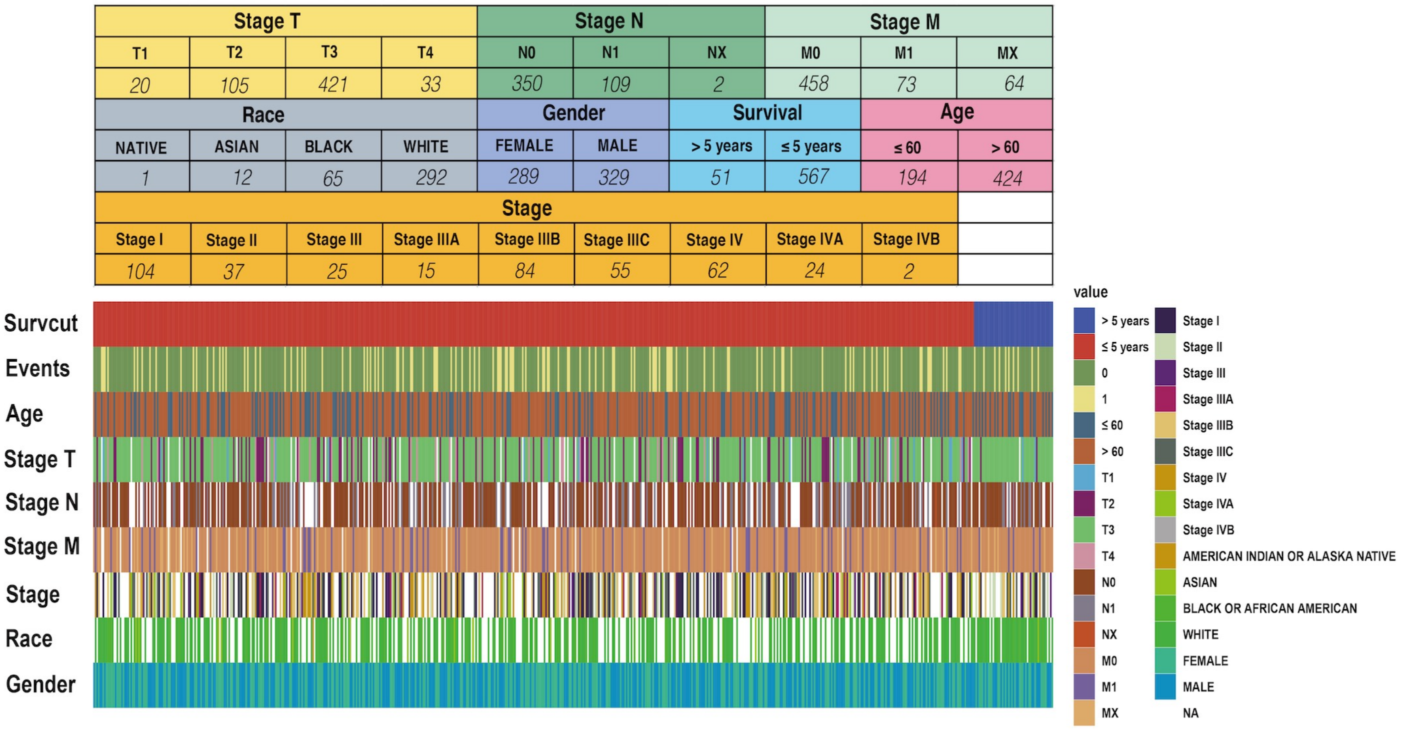

Figure S1: The clinical and pathology information of TCGA samples.

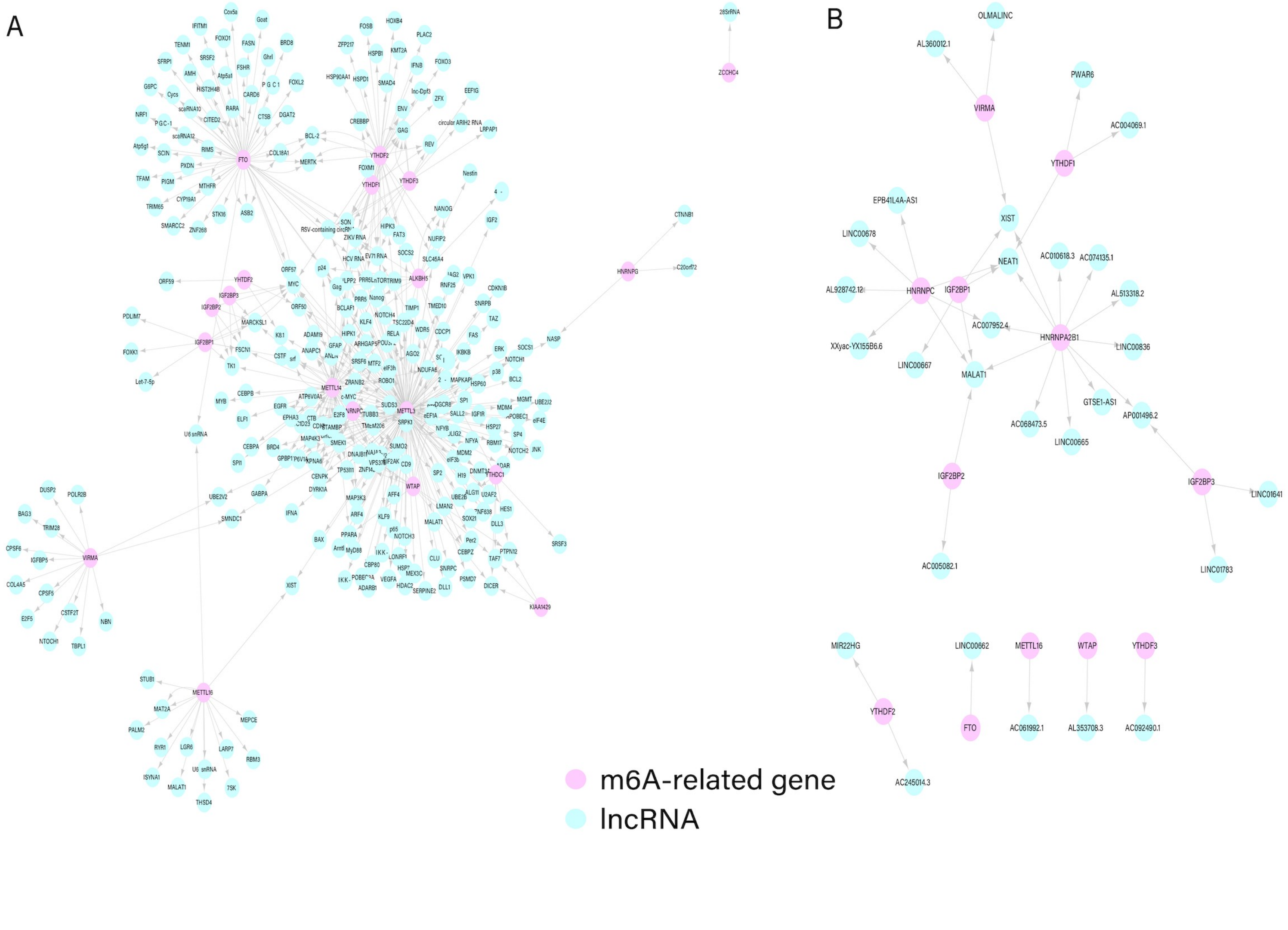

Figure S2: Networks of m6A-related lncRNAs. (A) lncRNAs obtained from the m6A2Target database. (B) lncRNAs obtained from the starBase database.

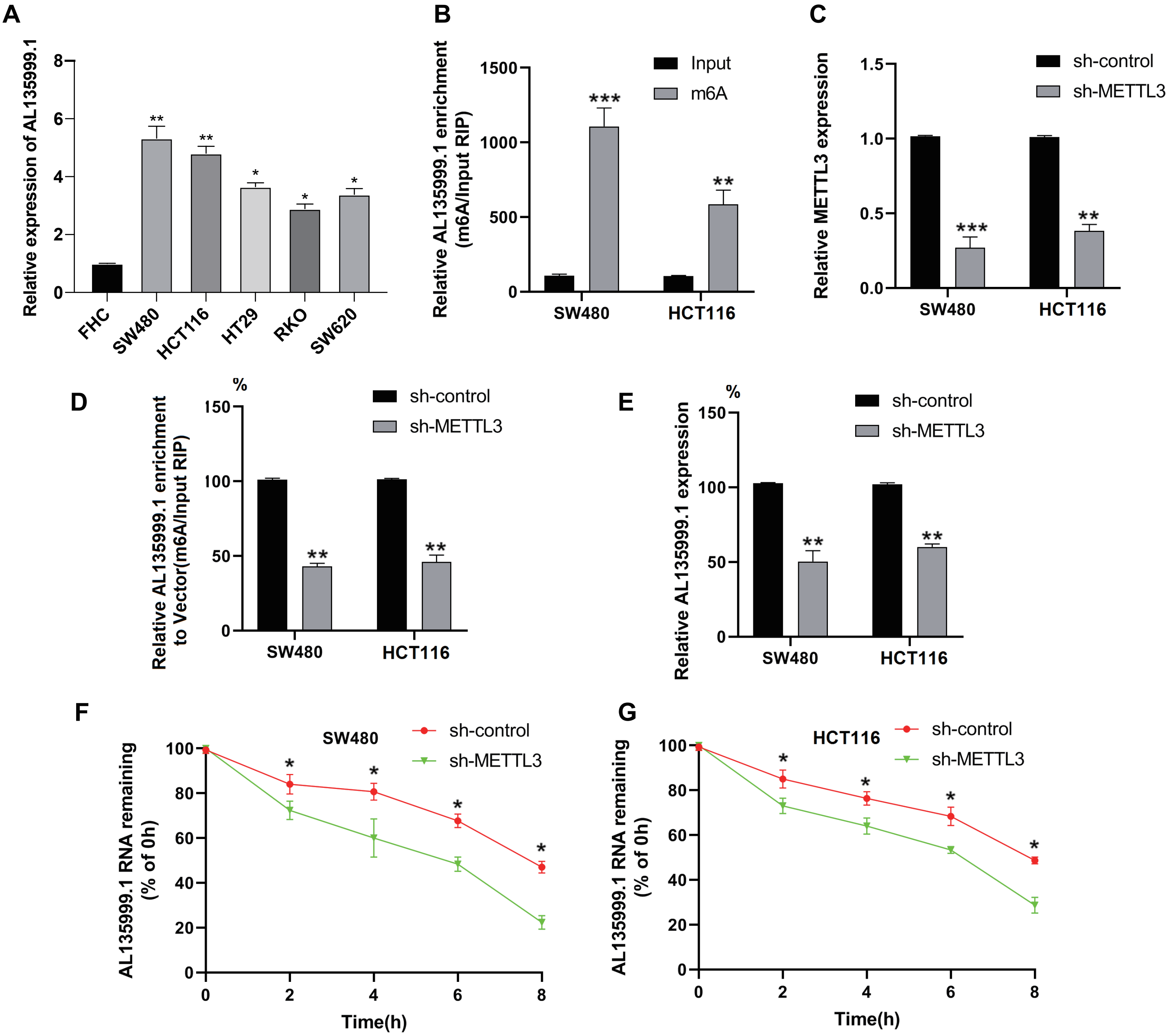

Figure S3: Upregulation of AL135999.1 in colorectal cancer is related to m6A modification. (A) Relative expression levels of AL135999.1 in different cells. (B) m6A modification is highly enriched in AL135999.1 of SW480 and HCT116 cells. (C) qRT-PCR confirmed the knockdown efficiency of METTL3. (D) Compared with the control group, the m6A level of AL135999.1 in SW480 and HCT116 cells with METTL3 knockdown. (E) The expression level of AL135999.1 was assessed in SW480 and HCT116 cells with METTL3 knockdown. (F) Treat SW480 cells of METTL3 knockdown with actinomycin D, and detect the expression level of AL135999.1 by qRT-PCR. (G) Treat HCT116 cells of METTL3 knockdown with actinomycin D, and detect the expression level of AL135999.1 by qRT-PCR.

Table S1. Primer sequence

Target	Primer sequence
GAPDH	F: 5’-CGGATTTGGTCGTATTGGG-3’
	R: 5’-CTGGAAGATGGTGATGGGATT-3’
METTL3	F: 5’-GTGTCGGAGGTGATTCCAGT-3’
	R: 5’-CTGCGCATCTCATCATCTGT-3’
AL135999.1	F: 5’-GCGATCAGTTTCAGGCACTC-3’
	R: 5’-TTGATGAAGGCTAGTGGGGC-3’
AL049840.4	F: 5’-TCATGTAACAGGGTGGGTGG-3’
	R: 5’-GACATGAGGCGGGATGAAAC-3’
